# Cardiopulmonary exercise capacity and quality of life of patients with heart failure undergoing a functional training program: study protocol for a randomized clinical trial

**DOI:** 10.1186/s12872-020-01481-6

**Published:** 2020-04-25

**Authors:** Daniela Meirelles do Nascimento, Karina Costa Machado, Patrícia Martins Bock, Marco Aurélio Lumertz Saffi, Livia Adams Goldraich, Anderson Donelli Silveira, Nadine Clausell, Beatriz D. Schaan

**Affiliations:** 1 Exercise Pathophysiology Laboratory, Hospital de Clínicas de Porto Alegre, Porto Alegre, RS Brazil; 2 National Institute of Science and Technology for Health Technology Assessment (IATS) – CNPq/Brazil, Hospital de Clínicas de Porto Alegre, Clinical Research Center, Rua Ramiro Barcelos, Porto Alegre, RS 2350 Brazil; 3 Faculdades Integradas de Taquara, Taquara, RS Brazil; 4 London Health Sciences Center and Western University, London, Canada; 5 Medical School, Universidade Federal do Rio Grande do Sul, Porto Alegre, RS Brazil

**Keywords:** Heart failure, Exercise, Functional training, Cardiopulmonary exercise capacity, Quality of life

## Abstract

**Background:**

Exercise intolerance is a common finding in heart failure that generates a vicious cycle in which the individual starts to limit his activities even more due to progressive fatigue. Regular physical exercise can increase the cardiopulmonary exercise capacity of these individuals. A new approach to physical exercise, known as functional training, could improve the oxygen consumption and quality of life of patients with heart failure; however, there is no information about the effect of this modality of exercise in this patient population. This randomized trial will compare the effects of 36 sessions of functional training versus strength training in heart failure patients.

**Methods:**

This randomized parallel-design examiner-blinded clinical trial includes individuals of both sexes aged ≥40 years receiving regular follow-up at a single academic hospital. Subjects will be randomly allocated to an intervention group (for 12-week functional training) or an active comparator group (for 12-week strength training). The primary outcomes will be the difference from baseline to the 3-month time point in peak oxygen consumption on cardiopulmonary exercise testing and quality of life assessed by the Minnesota Living with Heart Failure Questionnaire. Secondary outcome measures will include functionality assessed by the Duke Activity Status Index and gait speed test; peripheral and inspiratory muscular strength, assessed by hand grip and manovacuometry testing, respectively; endothelial function by brachial artery flow-mediated dilation; lean body mass by arm muscle circumference; and participant adherence to the exercise programs classified as a percentage of the prescribed exercise dose.

**Discussion:**

The functional training program aims to improve the functional capacity of the individual using exercises that relate to his specific physical activity transferring gains effectively to one’s daily life. In this context, we believe that that functional training can increase the cardiopulmonary exercise capacity and quality of life of patients with heart failure. The trial has been recruiting patients since October 2017.

**Trial registration:**

NCT03321682. Registered on October 26, 2017.

## Background

Exercise intolerance is a common finding in heart failure (HF) that generates a vicious cycle in which the individual starts to limit his activities even further due to progressive fatigue [1]. The mechanisms underlying exercise intolerance are generally considered multifactorial and include endothelial dysfunction. In patients with HF, improved endothelial dependent dilation as a result of exercise training is associated with increased exercise capacity, even in the absence of improved cardiac output [2]. Patients with HF, even when stable and compensated, experience a decline in functional capacity associated with a lower quality of life [3, 4]. Recent evidence suggests that exercise-based cardiac rehabilitation improves quality of life and functional capacity [5]. Despite the known benefits of physical training for patients with HF, the rates of adherence with recommended exercise are low, potentially limiting its ability to improve clinical outcomes [6].

In fact, a loss of strength and muscle mass, known as sarcopenia, is highly prevalent in this population and constitutes an important determinant of functional independence, hospitalization rates, and quality of life [7]. Patients with HF have a 30% decreased ability to perform activities of daily living (ADLs) compared to healthy individuals, which has been attributed to reduced muscle mass and decreased oxygen consumption (VO_2_) [8]. In this context, strength training increases muscle torque and endurance, functional independence, and quality of life, reducing the morbidity of individuals with and those without cardiovascular disease [9]. The lower overload to the cardiorespiratory system related to strength training may be a safe and comfortable alternative for exercise prescription to patients with HF [10]. Recent meta-analyses have shown a significant increase in peak VO_2_ in patients with HF as a clinical outcome of strength training [11, 12].

However, ADLs require a combination of endurance and strength, and aerobic training alone does not improve muscle strength [13]. In addition, traditional resistance/strength training does not ideally represent the movements performed during ADLs since it does not include exercises using coordinated and multiplanar movement patterns or incorporate multiple joints and dynamic tasks [14].

**Fig. 1:**
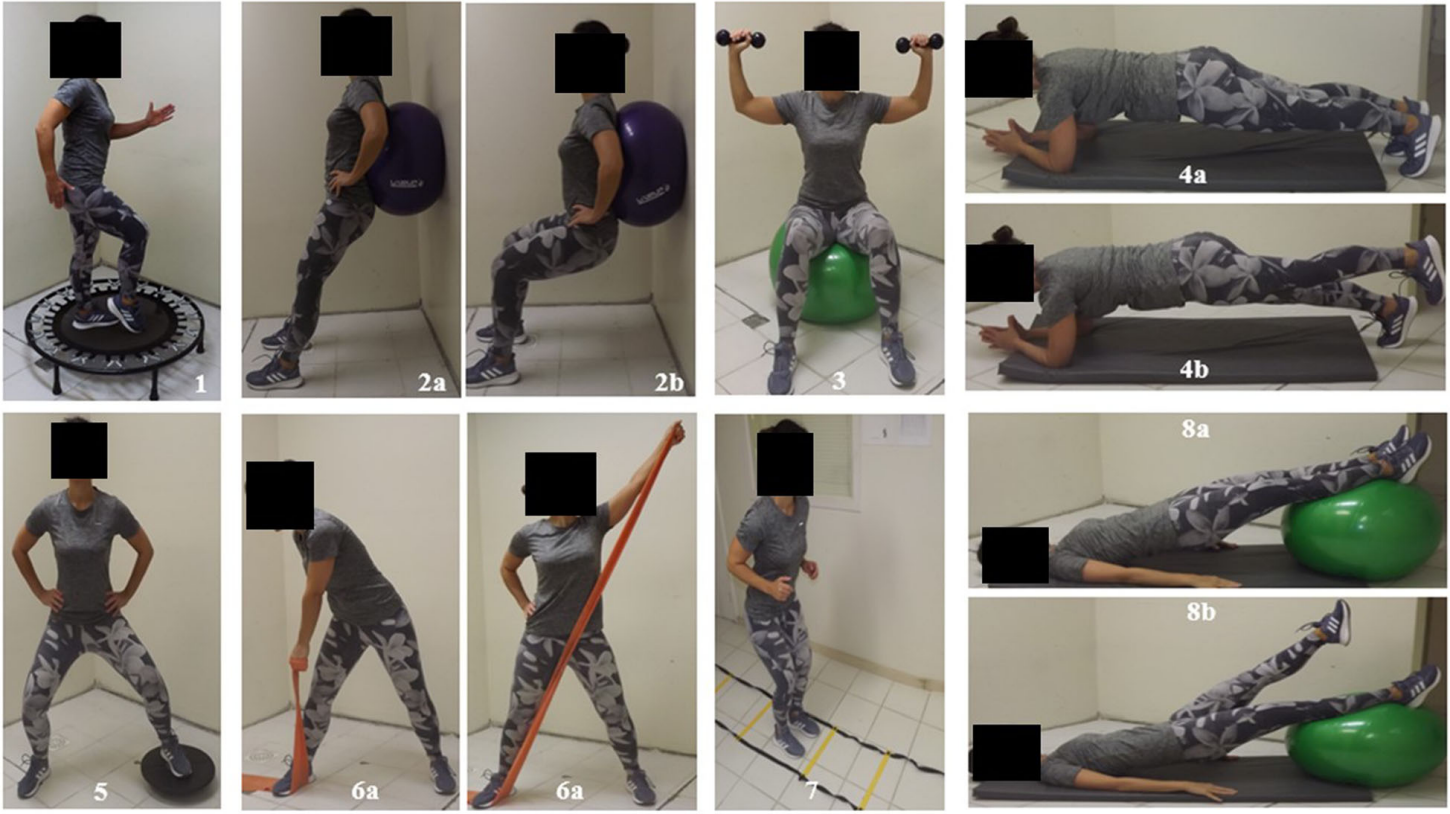
Examples of functional exercises. Legend: 1, run on trampoline; 2 (a, b), Swiss ball wall squat; 3, overhead press with dumbbells; 4 (a, b), plank and variation; 5, squat on rigid balance board; 6 (a, b), oblique twist with elastic band; 7, agility on ladder drills; 8 (a, b), reverse plank

Functional training may be a potential effective nonpharmacological therapeutic intervention for patients with HF. Articles related to functional training published to date did not include peak VO_2_ as an outcome, which makes this intervention innovative for patients with HF. Studies including functional training basically focused on assessing functionality in an elderly population in terms of walking capacity [15] and mobility [16]. This exercise method consists of integrated movements of the body in several planes that involve joint acceleration and deceleration, stabilization, strength, and neuromuscular efficiency (Fig. 1).

The method aims to improve the functional capacity of the individual using exercises that relate to his specific physical activity, effectively transferring gains to one’s daily life [14, 17]. In fact, functional training, sometimes called neuromotor exercise training, is a recommendation of the American College of Sports Medicine for apparently healthy adults of all ages [18]. Functional training could improve peak VO_2_ since it also includes an aerobic component. However, some aspects such as volume, performance patterns, and progression remain unknown. Additionally, the effectiveness of exercise training in chronic diseases has not been established [14]. The choice of the strength group as an active comparator group was because this type of training does not increase peak VO_2_ in the same magnitude as exercises with a larger cardiopulmonary component, as we expect in functional training. The primary objective of the present study is to compare the effects of functional training versus strength training on the cardiopulmonary exercise capacity and quality of life of patients with HF. Secondary objectives are to evaluate the: (1) effects of functional training versus strength training on functionality, muscle strength, endothelial function, and lean body mass; (2) adherence of participants to both physical training protocols; and (3) safety of a functional training program.

## Methods/design

### Study setting

This randomized parallel-design 1:1 ratio allocation examiner-blinded clinical trial is conducted at the Hospital de Clínicas de Porto Alegre (HCPA), a tertiary hospital in the city of Porto Alegre, Southern Brazil. The study was approved by the facility’s institutional review board on August 8, 2017 (protocol no. 20170291). The study protocol adheres to the SPIRIT 2013 recommendations [19] (Additional file 1). The World Health Organization Trial Registration Dataset is provided herein (Additional file 2). Written informed consent is obtained from all patients before participation (Additional file 3).

### Eligibility criteria

The study’s inclusion and exclusion criteria are defined below.

#### Inclusion criteria

- Age equal to or older than 40 years.
- Clinically stable HF (ischemic and non-ischemic) for at least 3 months before randomization and diagnosed according to clinical records.
- New York Heart Association (NYHA) functional class II–III with slight to marked limitation of physical activity, respectively [20].
- Left ventricular ejection fraction (LVEF) equal to or less than 45%.
- Optimized pharmacological treatment [20].

#### Exclusion criteria

- Enrollment in another clinical trial involving physical training protocols.
- Regular practice of physical exercise of more than 150 min per week [21] in the last 3 months.
- Decompensated HF.
- Acute myocardial infarction and/or cardiac surgery for less than 6 months.
- Severe valvular heart diseases and/or uncontrolled cardiac arrhythmias.
- Asymmetric septal hypertrophic cardiomyopathy with a dynamic obstruction in the outflow pathway.
- Musculoskeletal disorders limiting completion of the protocol exercise program.
- Impaired cognitive status that compromises the understanding of the steps and completion of the study protocol.

### Intervention

The participants are randomly allocated to a functional training program or a strength training program, each lasting 3 months. The exercise sessions are completed at the Center of Clinical Research at the HCPA. The patients perform the exercise training three times/week for a total of 36 sessions. Both physical exercise training programs are performed under the supervision of a physiotherapist who specialized in sports sciences. The exercises are performed individually or in pairs. Resting heart rate (HR) and blood pressure are measured with a validated digital automatic sphygmomanometer before and after each training session. The first two training sessions are earmarked for patients to become familiar with the exercises.

Each training session lasts approximately 50–60 min, consisting of an initial warm-up of 5 min, 35–45 min of functional or strength exercises, and 10 min of stretching and cool-down (Table 1).

**Table 1:** Phases of the sections and collection of exercises performed in training protocols

Functional Training Session	Strength Training Session	Time
***Warm-up:*** Run on the trampoline	***Warm-up:*** Stationary gait and calisthenics exercises	5 min
***Functional exercises:*** Core strength Agility and balance Knee and hip dominance Vertical pressure Horizontal pressure Horizontal and vertical pulling	***Strength exercises:*** Shoulder abduction Triceps extension Biceps curls, bench press Abdominals, calf raises Leg extension, seated leg curl Leg press and leg abduction	35–45 min
***Cool-down:*** Stretching exercises for lower limbs, upper limbs, and spine	***Cool-down:*** Stretching exercises for lower limbs, upper limbs, and spine	10 min

#### Functional training

Each phase of functional training includes a total of 10–12 different exercises. Prescribed movements consist of multi-joint exercises emphasizing major muscle groups and ADLs like sitting, standing up, pushing, and pulling. Unstable surfaces, cones, ladder drills, elastic bands, kettlebells, dumbbells, steps, and Swiss balls are used in the training. Exercise intensity is self-paced, although the participants are encouraged by the physical therapist to exercise at high performance (time and number of repetitions) and progressively increase its performance or the difficulty of each exercise. A rest period between the series is determined by the time required for proper patient positioning and breath recovery. Indeed, the exercises are adjusted for each session depending on changes in functional and health status. The sequence of the functional exercises is designed to alternate strength exercises with agility training or strength exercises with aerobic conditioning as well as arm exercises with leg exercises always grouped in pairs or every three exercises. Running on the trampoline is performed as the warm-up and repeated at the end of the session before stretching exercises (Table 2).

**Table 2:** Functional training protocol: exercises and periodization model

**Phase 1** − 4 weeks-	Run on trampoline	–	3 sets of 40 s
	Sit to stand (body weight)	–	2 sets of 30 s
	Biceps curls with elastic band	–	2 sets of 10–12 rep
	Suicide	–	2 sets of 30 s
	Sumo squat (with 4-kg kettlebell)	–	2 sets of 30 s
	Standing band row	–	2 sets of 10–12 rep
	Low step up (10-cm height)	–	2 sets of 40 s
	Lateral band walk	–	2 sets of 30 s
	Basic crunch	–	2 sets of 10–20 s
	Hip adductor ball squeeze	–	2 sets of 30 s
	Frontal plank	–	2 sets of 15–20 rep
	Run on trampoline	–	3 sets of 40 s
**Phase 2** − 4 weeks-	Run on trampoline	–	3 sets of 50 s
	Squat on rigid balance board	–	1 set of 30 s for each side
	Oblique twist with elastic band	–	1 set of 10–12 rep for each side
	Side to side run with cones	–	1 to 2 sets of 30 s
	Kettlebell high pull (4 kg)	–	2 sets of 10–12 rep
	Lunges (body weight)	–	1 to 2 sets of 30 s for each leg
	Low step up (10-cm height)	–	2 sets of 50 s
	Basic crunch	–	2 sets of 15–20 rep
	Reverse crunch	–	1 to 2 sets of 15–20 rep
	Swiss ball hip raise	–	1 set of 10 rep
	Frontal plank	–	1 to 2 sets of 10–20 s
	Lateral plank	–	1 set of 10–20 s
	Run on trampoline	–	3 sets of 50 s
**Phase 3** − 4 weeks-	Run on trampoline	–	3 sets of 60 s
	Swiss ball wall squat	–	2 sets of 30 s
	Triceps bench dips	–	2 sets of 10–12 rep
	Step up (20-cm height)	–	1 set of 30 s for each leg
	Agility on ladder drills	–	1 to 2 sets of 30 s
	Dumbbell shoulder press Swiss ball (2 kg)	–	2 sets of 10–12 rep
	Walking lunge (body weight)	–	1 to 2 sets of 30 s
	Basic crunch	–	2 sets of 15–20 rep
	Reverse crunch	–	1 to 2 sets of 15–20 rep
	Hamstring curl with a Swiss ball	–	2 sets of 10–12 rep
	Reverse plank with leg lift	–	4 sets of 3 s for each leg
	Plank with leg lift	–	1 set of 10 s for each leg
	Run on trampoline	–	3 sets of 60 s

*Sec* second, *Rep* repetition

#### Strength training

The strength training follows the recommendations for resistance training in individuals with cardiovascular disease by the American Heart Association [9]. The exercise protocol involves large muscle groups alternating their execution between the upper and lower limbs. Free weights (dumbbells, barbells, and ankle weights) and weight machines are used in the training. Two sets of 8–12 repetitions for the upper limbs and 12–15 repetitions for lower limbs are performed. The participants are encouraged by the physical therapist to perform a higher number of repetitions. The first two sessions are designed to determine the load of each exercise using the Borg scale (effort target of 3–4) and adapt the participant to the training. The progression of the exercises occurs at 4-week intervals.

#### Criteria for discontinuation and safety interventions

A participant may be discontinued from the study at the investigator’s discretion for safety reasons. For any study subjects, an incident cardiovascular event, hospitalization, or severe health event during the intervention period are considered criteria to discontinue study participation. If typical thoracic pain, disabling dyspnea, and/or exercise-related syncope occur during the training session, the exercise intervention will be interrupted. Trained nursing staff at the clinical research center will provide clinical assessment and event-directed interventions accordingly. If necessary, immediate transfer to the hospital’s emergency department will be performed.

#### Strategies for trial retention

During weekends, participants allocated to both groups receive phone calls to reinforce intervention session time and place. We use phone calls to inquire about any adverse events if a participant misses a session of any intervention arm. The phone call schedule will cease for participants declaring their withdrawal from the study.

### Outcomes

**Fig. 2:**
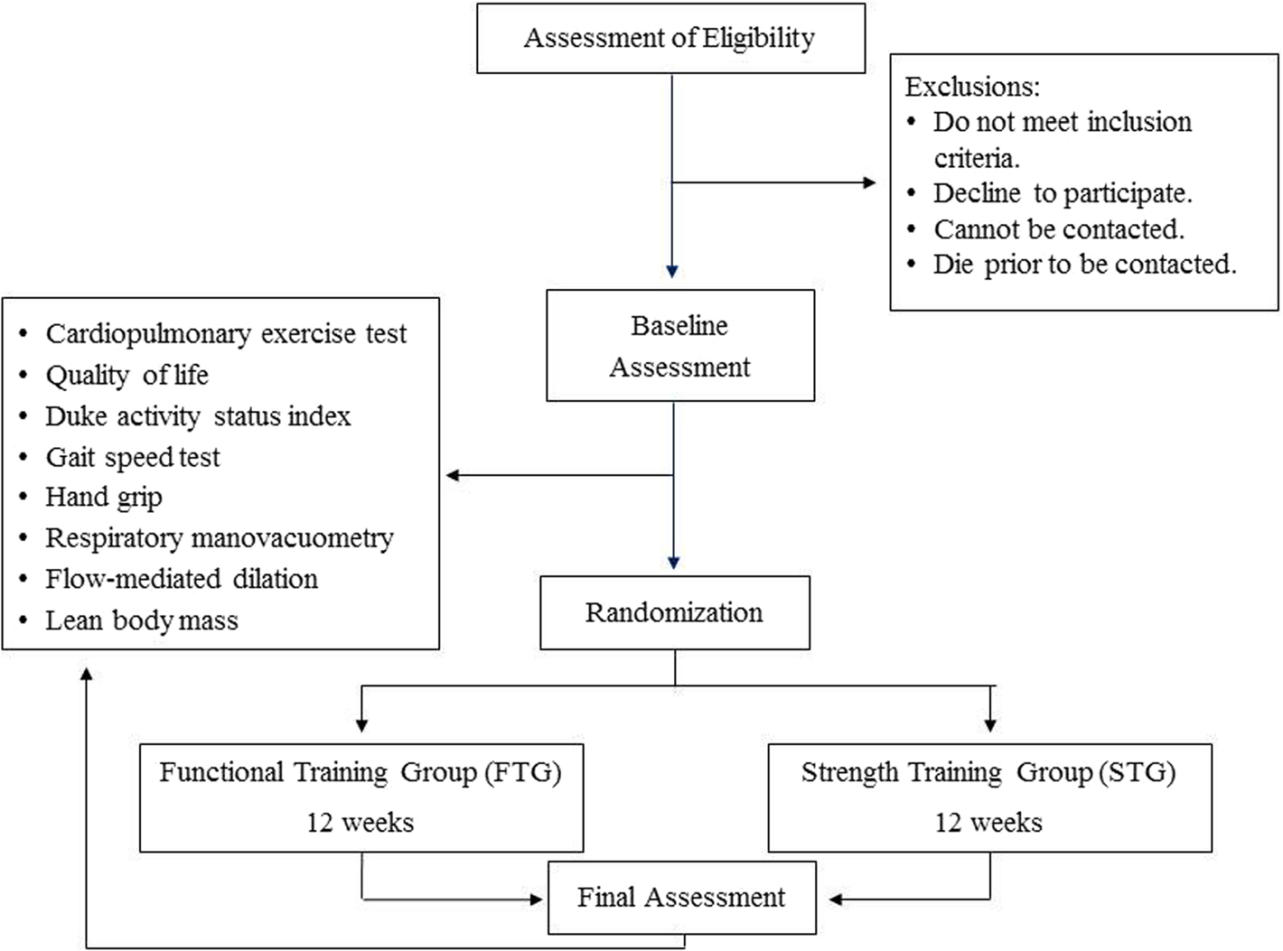
Proposed trial design

The outcomes are assessed at baseline and after the provision of written informed consent but before allocation to one of the study groups. At the end of the 12-week period, the subject will be re-evaluated (Fig. 2).

Patients’ baseline demographic and clinical information including age, sex, HF etiology, left ventricle ejection fraction, and NYHA functional class are obtained from their electronic health records.

The primary outcome measures are cardiopulmonary exercise capacity and quality of life; the secondary outcomes include assessment of functionality, peripheral and inspiratory muscle strength, endothelial function, lean body mass, and participant adherence to exercise programs.

### Measurement of primary outcomes

#### Cardiopulmonary exercise capacity

Cardiopulmonary exercise testing with expired gas analysis is performed on a treadmill (T2100, General Electric, WI, USA; speed 0–22 km/h [0–13.5 mph], grade 0–26%). A ramp protocol is used with a starting speed of 2.0 km/h or 2.5 km/h and a starting grade of 0%. Increments of 0.5 km/h per minute in speed and 1% per minute in grade were used to achieve fatigue within 8–12 min. During the test, gas exchanges are continuously measured breath-by-breath by a previously validated system (Quark CPET; COSMED, Rome, Italy).

Blood pressure is monitored every 3 min using a sphygmomanometer. HR is monitored using 12-lead electrocardiography (Quark C12x; COSMED) with electrode placement as described by Mason and Likar [22]. The test analyzes the ventilatory and metabolic variables. Peak VO_2_ is set to the highest 20-s average value reached during the test. Maximality criteria are defined by a respiratory exchange ratio greater than or equal to 1.05.

#### Quality of life

The Minnesota Living with Heart Failure Questionnaire, a disease-specific instrument used to assess quality of life, consists of 21 questions about limitations that are often associated with how HF prevents patients from living as they would like to. Patients answer the questions as they related to the previous month. The total score is 0–105 points. A low score reflects a better health-related quality of life. The instrument is validated in Portuguese [23].

### Measurement of secondary outcomes

#### Functionality

The Duke Activity Status Index is a 12-item questionnaire that assesses daily activities. Each item has a specific weight based on metabolic equivalents. The participants identify each of the activities that they can perform. The final score is 0–58.2 points. A higher score represents better functional capacity. The instrument is validated in Portuguese [24].

The gait speed test is used to evaluate and monitor the functional status and general health of a wide variety of populations. The method requires a 20-m corridor. The patient walks at his own pace, without running, and the time spent in the central 10 m of the corridor is determined. The ratio between distance and time (meters/second) will then be calculated [25].

#### Peripheral muscle strength

The hand grip dynamometer has been widely used to evaluate the nutritional status, functional, lateral dominance, and total strength of individuals always divided into groups by sex and age. Hand grip strength is assessed as recommended American Association of Hand Therapists [26] using a JAMAR® dynamometer (Sammons Preston, Inc., Bolingbook, IL, USA). The strength values in kilograms ​​will be calculated as the average of three attempts for the dominant hand performed at 1-min intervals between measurements.

#### Inspiratory muscle strength

Manovacuometry assesses inspiratory muscle strength or maximal inspiratory pressure (MIP) by maintaining a maximum negative pressure for at least 1 s after a forced expiration to residual volume against an occluded airway as recommended by the American Thoracic Society. At least three reproducible maneuvers must be performed using a digital pressure manometer (MVD300; Globalmed, Porto Alegre, Brazil). For the data analysis, the highest value is recorded if it does not exceed the second highest value by 10% [27].

#### Endothelial function

Noninvasive measurements of endothelial function are obtained by flow-mediated dilation of the brachial artery using two-dimensional ultrasonography. Its measurement is performed in accordance with published guidelines [28] always by the same trained operator (MALS). Briefly, the examination starts after a 15-min rest in a temperature-controlled room with the patient supine and the arms in a comfortable position. Any vasodilators are discontinued at least 4 h before the examination if possible. The individuals are advised to refrain from exercising, drinking caffeine, and smoking for at least 4 h before the examination.

#### Lean body mass

The arm muscle circumference is obtained from arm circumference and tricipital skinfold measurement using a tape measure and an adipometer, respectively [29].

#### Adherence

Adherence to an exercise program has been classified as meeting at least 80% of the recommended or prescribe exercise dose. Any participant who demonstrates a training protocol adherence of 80% or more than 36 sessions will be considered adherent. Participants will be classified as non-adherent or partially adherent if their adherence is less than 80% [30].

### Sample size

Our sample size calculation was based on Feiereisen et al., who enrolled subjects with HF with reduced ejection fraction to assess the effect of strength in comparison with aerobic and combined aerobic-strength training on their peak VO_2_ [31]. Considering a power of 80%, a significance level of 5% and an effect size of 0.2 for the peak VO_2_, a total sample size of 32 subjects was estimated, including 16 in each study group. To account for a 20% estimated participant loss or refusal rate, we defined that 19 patients should be enrolled in each group, totalizing 38 subjects.

### Recruitment

The patients are recruited from the outpatient HF clinic of Hospital de Clínicas de Porto Alegre. The recruitment period for the study is planned to range from October 2017 to July 2020. Eligible patients are informed of the study and invited to participate; those who accept are tested by a blinded assessor and randomly allocated to one of the study groups. A flow diagram of the patient recruitment process is shown in Fig. 2.

### Assignment of interventions and blinding

Group allocation was determined by eight blocks of 4 individuals (Software Rx64 version 3.1.1) in a 1:1 ratio generated by an external researcher. The investigator in charge of randomization does not participate in the other data collection stages. Allocation concealment is implemented through a central randomization routine conducted by investigators with access to the randomized list and the investigator charged with requesting the code to place subjects in the intervention group. In brief, the assigner contacted the external researcher to request whenever one or more subjects should enter an intervention arm. Thereafter, the external researcher will consult the code in consecutive order and uncover the code relative to the requested subject(s). Such requests will be documented and archived for further accountability. To ensure intervention blinding, communication with participants is not performed by the investigators involved in the outcome assessments.

Due to the nature of the interventions, the researcher conducting the exercise sessions as well as participants are not blinded. To ensure assessor masking, the subjects are asked to omit their assigned group and not to talk about their interventions during the outcome evaluation sessions. In the case of unintentional unblinding for any reason, the involved researcher will notify the principal researcher. In such cases, participant ID, date, and unblinding circumstances will be documented for internal control.

### Data collection

Standard operating procedure documents are available for each assessment. The outcome assessors were trained and the handling of a standard operating procedure short version is mandatory during each data collection period. All variables will be assessed at baseline (prior to randomization) and at study completion.

### Statistical analysis

The characterization of the sample will be performed by descriptive statistical analysis using measures of central tendency (mean and median) and variability (standard deviation and interquartile range). The normality of the data will be tested by the Shapiro-Wilk test. Intra- and inter-group analyses will be performed before and after the total 12-week intervention period. For both analyses the generalized estimation equations (GEE) will be used. Correlations between peak VO_2_ and study variables – quality of life, functionality, muscle strength, endothelial function, and lean body mass – will be examined by Pearson or Spearman coefficients as appropriate.

In all tests, a significance level of *p* < 0.05 will be adopted. All data will be analyzed using SPSS Statistics for Windows version 20.0 (IBM Corp., Armonk, NY, USA) by the intention-to-treat and protocol methods.

Dropouts (essentially, participants who withdraw consent for continued follow-up) or missing data will be included in the analysis by modern imputation methods.

### Monitoring

#### Data monitoring

The study does not have a data monitoring committee. We reason that this committee would not be mandatory due to the characteristics of the interventions and outcomes despite the trial’s high overall quality.

#### Harms

The study will monitor for the following physical training-related adverse effects during the intervention period: shortness of breath, fatigue, and muscular pain. The researcher responsible for the intervention will identify possible solutions for any adverse effects.

#### Auditing

If necessary, auditing will be conducted by the Hospital de Clínicas de Porto Alegre using defined protocols implemented by an independent monitoring team adjunct to the research board structure.

## Discussion

Thirty-five percent of HF patients die within 5 years after diagnosis, and this syndrome remains the major cause of hospitalization for patients older than 65 years of age. Thus, its impact in the health care systems is high [32].

The present study is the first to evaluate the effect of functional training in HF patients. This modality emerged from the training of athletes and the rehabilitation of sports injuries and lower back pain [17]. It was mainly studied in elderly populations with a focus on reducing the fall risk and late-life disability [14–16].

Compared to a moderate-intensity walking program, a functional circuit training program performed at high intensity for 6 weeks by sedentary subjects significantly improved their maximum leg and shoulder strengths. The maximum cycling workload evaluated on a bicycle ergometer was also significantly higher in the functional training group, whereas maximal VO_2_ and ventilatory threshold were not [33]. In fact, this study enrolled a healthy and young population (mean age, 25 ± 5 years old) constantly exercised at submaximal intensity. It is possible that functional training could lead to a more expressive improvement in cardiorespiratory parameters when performed by individuals with lower physical fitness and some degree of disability, such as those with HF.

Exercise intolerance is a hallmark symptom of HF and associated with increased disability and mortality [34]. The sedentary lifestyle adopted by individuals with HF leads to peak VO_2_ reductions and poor quality of life [35]. Although VO_2_ is an important prognostic predictor of HF, its increase is not related to improvement in left ventricular ejection fraction, and a recovery of central hemodynamic function does not translate to improved exercise performance [36]. On the other hand, VO_2_ is significantly correlated with quadriceps muscle mass, mean arm circumference, and muscle area, suggesting that atrophy of the peripheral muscles contributes to exercise intolerance in patients with HF [37]. Skeletal muscle strength is strongly correlated with morbidity and mortality of patients with HF and an independent predictor of peak VO_2_ [9].

Different combinations of aerobic (continuous and interval), strength/resistance, and inspiratory exercise training have been proposed to patients with HF [38]. Laoutaris et al. [37] randomized 27 patients to a 12-week aerobic or a combined aerobic, resistance, and inspiratory muscle training program. The combined protocol demonstrated a significantly greater increase in quadriceps strength and resistance than aerobic training alone. However, the increase in peak VO_2_ and MIP were similar in both groups. Dall’Ago et al. [39] evaluated the effects of inspiratory muscle training in inspiratory muscle strength and functional capacity in patients with HF and inspiratory muscle weakness. After the 12-week training period, the patients in the intervention group demonstrated significantly improved functional capacity as evidenced by an increased 6-min walk distance and peak VO_2_ versus the placebo group. Quality of life was also significantly increased in the trained group. Maiorana et al. [40] randomized 36 untrained subjects with HF to 12 weeks of resistance training, aerobic training, or an untrained control group. Peak VO_2_ increased after 12 weeks of aerobic training and 6 and 12 weeks of resistance training but decreased in controls at 12 weeks.

The diversity of training protocols makes it difficult to generalize the findings cited above and the question remains open: Is there a complete protocol for physical training for patients with HF?

It is important that we investigate the benefits of functional training that mimics the daily activities with a combination of resistance/strength muscle and aerobic exercise.

## Supplementary information

**Additional file 1.** SPIRIT 2013 Checklist: Recommended items to address in a clinical trial protocol and related documents*.

**Additional file 2.** World Health Organization Trial Registration Dataset.

**Additional file 3.** Consent form.

## Data Availability

We support the reuse of scholarly data and intend that the data to be collected in this trial may contribute beyond our actions to the knowledge on exercise and non-pharmacological management of HF. First, we will provide in writing the final results of the research for each participant. Second, we have obtained ethical consent from participants as well as research ethics board approval to share deidentified data after trial completion through presentation in congresses and publications in journals.

## Abbreviations

ADL: Activities of daily living
HF: Heart failure
HR: Heart rate
LVEF: Left ventricular ejection fraction
MIP: Maximal inspiratory pressure
NYHA: New York heart association
VO_2_: Oxygen consumption
