# Supplementary material for: Cardiopulmonary exercise capacity and quality of life of patients with heart failure undergoing a functional training program: study protocol for a randomized clinical trial
Source: BMC Cardiovasc Disord. 2020 Apr 25;20:200. doi: 10.1186/s12872-020-01481-6 (PMC7183632; doi:10.1186/s12872-020-01481-6)
Supplement: Supplementary file 2 — Additional file 2. World Health Organization Trial Registration Dataset. [file 12872_2020_1481_MOESM2_ESM.docx]

**Additional file 2**

**World Health Organization Trial Registration Dataset**

1. **Primary Registry and Trial Identifying Number**
   ClinicalTrials.gov, NCT03321682
2. **Date of Registration in Primary Registry**October 26^th^, 2017.
3. **Secondary Identifying Numbers**
   Federal IRB Number: CAAE 69314017.8.0000.5327

WebGPPG Number: 20170291

1. **Source(s) of Monetary or Material Support**

FIPE (Fundo de Incentivo à Pesquisa e Eventos, Hospital de Clinicas de Porto Alegre)

1. **Primary Sponsor**
   Hospital de Clínicas de Porto Alegre
2. **Contact for Public Queries**
   Beatriz Schaan, PhD, MD, ScD
   Hospital de Clínicas de Porto Alegre
   Rua Ramiro Barcelos, 2350 - Hospital de Clínicas de Porto Alegre, Porto Alegre, RS, Brazil
   Ph: +55 51 3359.8276
   Email: bschaan@hcpa.edu.br
3. **Contact for Scientific Queries**
   Daniela Meirelles do Nascimento, MSc
   Hospital de Clínicas de Porto Alegre
   Rua Ramiro Barcelos, 2350 - Hospital de Clínicas de Porto Alegre, Clinical Research Center, 21301
   Porto Alegre, RS, Brazil
   Ph: +55 51 3359.6332 or +55 51 99971-4276
   Email: [dnascimento@brturbo.com.br](mailto:dnascimento@brturbo.com.br)
4. **Scientific Title**

Cardiopulmonary exercise capacity and quality of life of patients with heart failure undergoing to a functional training program – a randomized clinical trial

1. **Countries of Recruitment**

Brazil

1. **Health Condition(s) or Problem(s) Studied**

Heart failure

1. **Intervention(s)**

Experimental group: Functional training

Active comparator group: Strength training

1. **Key Inclusion and Exclusion Criteria**

- Inclusion criteria: Age equal or higher than 18 years; diagnosis of clinically stable, ambulatory HF for at least 3 months before randomization; New York Heart Association (NYHA) functional classes II-III; left ventricular ejection fraction (LVEF) equal or less than 45%; optimized pharmacological treatment; sedentarism.
- Exclusion criteria: Enrollment in another clinical trial involving physical training protocols; decompensated HF and/or metabolic diseases; acute myocardial infarction and/or cardiac surgery for less than six months; severe valvular heart diseases and/or uncontrolled cardiac arrhythmias; asymmetric septal hypertrophic cardiomyopathy with dynamic obstruction in the outflow pathway; musculoskeletal disorders limiting the execution of the protocol exercise program; impaired cognitive status that compromise the understanding of the steps and the execution of the study protocol.

1. **Study Type**

Randomized clinical trial

1. **Date of First Enrollment**

March 6, 2017.

1. **Target Sample Size**

Thirty two participants.

1. **Recruitment Status**

Recruiting.

1. **Primary outcome(s)**

Cardiopulmonary exercise capacity and quality of life (time frame: 12 weeks of physical training).

1. **Key Secondary Outcomes**

Functionality, peripheral and inspiratory muscle strength, endothelial function, lean body mass and participants adherence (time frame: 12 weeks of physical training).
