# Supplementary material for: Cardiopulmonary exercise capacity and quality of life of patients with heart failure undergoing a functional training program: study protocol for a randomized clinical trial
Source: BMC Cardiovasc Disord. 2020 Apr 25;20:200. doi: 10.1186/s12872-020-01481-6 (PMC7183632; doi:10.1186/s12872-020-01481-6)
Supplement: Supplementary file 3 — Additional file 3. Consent form. [file 12872_2020_1481_MOESM3_ESM.docx]

**CONSENT FORM**

Protocol No. 20170291

Study title: Cardiopulmonary exercise capacity and quality of life of patients with heart failure undergoing a functional training program –a randomized clinical trial

This consent form is part of the informed consent process. It is designed to give you an idea of what this research study is about and what will happen to you if you choose to be in the study. You are being invited to participate in a research whose objective is to evaluate the effects of functional training on cardiopulmonary capacity and quality of life in patients with chronic heart failure. This research is being conducted by the laboratory of Physiopathology of Exercise (LaFIEx) of the Hospital de Clínicas de Porto Alegre (HCPA).

Functional training consists of a series of physical exercises that use some materials, such as medicine ball and elastic band. Cardiopulmonary capacity is the ability of the individual to perform his/her physical activities. Quality of life is the individual's perception of their position in life, their goals, expectations, standards and concerns.

If you agree to participate in the survey, you will be drawn to one of the two groups of the study, and you cannot choose which group to participate in. They are:

1) The group that will perform the functional training, with exercises like squat, push, pull and jump, lasting approximately 1 hour, 3 times a week, for a total time of 12 weeks;

2) The group that will perform the strength training, with exercises performed against a resistance, lasting approximately 1 hour, 3 times a week, for a total time of 12 weeks.

The exercise sessions will be held at the research center of the Hospital de Clínicas de Porto Alegre. The exercise sessions of the functional training and the strength training will be performed according to previous scheduling.

In addition, the following assessments will be carried out on the first day before starting physical exercises and after all sessions are completed, not coinciding with the training days:

a) Cardiopulmonary exercise testing on treadmill, lasting between 10 to 20 minutes;

b) Walking speed test, in which you will have to walk in a flat and straight corridor of 20 meters;

c) Answer a questionnaire about the life activities you can perform, such as bathing and dressing; as well as a questionnaire that asks about your quality of life, including questions about how much your heart problem compromises your sexual activity or causes you to get depressed;

d) Evaluation of muscle strength assessed when you squeeze with your dominant hand an object, for about 5 seconds, for 3 times;

e) Evaluation of inspiratory muscle strength assessed when you take a deep breath through the mouth by a mouthpiece connected to a device that measures the pressure performed during the breathing effort. The maximum breathing force should be maintained for 1 second and the maneuver repeated 3 times;

f) A cuff will be placed on your arm, similar to the blood pressure measurement procedure, and how much your brachial artery can increase its diameter will be evaluated by means of ultrasonography. This exam will take about 30 minutes and may cause discomfort by the pressure of the device in the arm;

g) The circumference of your arm will be measured with a measuring tape.

These assessments and questionnaires will be conducted at the Clinical Research Center and will take approximately 4 hours, not counting the training sessions that will be performed on another day.

The possible risks or discomforts consist of shortness of breath, fatigue or muscular pain during or after exercises sessions, and embarrassment when answering the questionnaires and the time destined for participation.

The possible benefits arising from your participation in the research consist of increasing the ability to perform exercises. If an improvement in cardiopulmonary capacity is identified, functional training will be offered at the end of the study for the participants who did not undergo this type of training, for the same period performed in the group that was benefited.

Your participation in the research is totally voluntary, that is, it is not mandatory. If you decide not to participate, or to opt out of participating and withdraw your consent, there will be no harm to the attendance you receive or may receive at the institution.

No payment is provided for your participation in the survey and you will have no cost with respect to the procedures involved.

In the event of any intercurrence or damage resulting from your participation in the research, you will receive all the necessary care, with no personal cost.

The data collected during the research will always be treated confidentially. The results will be presented jointly, without the identification of the participants, that is, their name will not appear in the publication of the results.

If you have any questions, you can contact the researcher in charge Dr. Beatriz D´Agord Schaan telephone (51) 3359-6332, with the researcher Daniela Meirelles do Nascimento, telephone (51) 99971-4276 or with the Research Ethics Committee of the Hospital de Clínicas de Porto Alegre (HCPA), telephone (51) 3359-7640, or on the 2nd floor of the HCPA , Room 2227, from Monday to Friday, from 8am to 17h.

This term is signed in two ways, one for the participant and the other for the researchers.

____________________________________

Participant name

____________________________________

Signature

____________________________________

Researcher who applied the term

______________________________________

Signature

Location and Date: _________________________________________

Adress: Rua Ramiro Barcelos 2350 / 3º andar, Centro de Pesquisas Clínicas - LaFiEx (Laboratório de Fisiopatologia do Exercício / Telefone: 3359-6332), Hospital de Clínicas de Porto Alegre.
